# Principle vs. Policy: Consistency vs. Expediency

**Published:** 1885-09

**Authors:** 


PUBLISHED MONTHLY AT

AUSTIN, TEXAS.

F. E. DANIEL, M. D., EDITOR AND PUBLISHER,

Subscription Price: Two Dollars per Annum in Advance.

Papers for publication in this Journal should be in the hands of the Editor by the 10th of the month preceding the month in which it is wished to appear; they must be original,--never having appeared in print elsewhere, and must be contributed exclusively to this Journal.

Contributions to the department of Original Articles are cordially invited from the profession of Texas, especially. What is most desired is Clinical reports of cases, essays and short practical articles on any medical topic ; also solicited reports of proceedings of societies, clubs, etc., and items of medical news of general interest to the profession, such as notices of appointments of physicians, removals, changes, marriages, deaths, etc.

### EDITORIAL.

### PRINCIPLE vs. POLICY.

### CONSISTENCY vs. EXPEDIENCY.

It does seem strange, that the profession of America, composed, as it is, of men of high intelligence, should be so divided upon a subject which, looked at dispassionately, is susceptible of but one construction. All this great fuss has been raised on the unwarranted assumption that "certain disappointed members dragged the code into the discussion of the International Congress question, at New Orleans." There never was a greater mistake.

The facts, as observed by one who took an active part in the discussion, are as follows:

The committee of eight, we'll say, believed they were empowered by the resolutions under which they were acting, to do as they did, organize the Congress; and that too, without further consulting the Association. At least they took that course, and instead of submitting to the Association an outline of what they proposed to do, they made the appointments and announced the organization of the Congress in the Journal of the Association, giving it, thereby, the appearance of being official, and done by the consent of the Association. This the Association construed into a breach of authority, and a downright discourtesy. The Association did not put the construction upon the resolution that the committee did, but regarded the committee as one of arrangements only; and when it transpired that they had assumed,and exercised plenary powers, and an entire independence of the source whence any power, whatsoever, was derived, there was left but one of two alternatives: to acquiesce and approve, or to protest.

Thus it will be seen, it was, primarily, a question of jurisdiction; the Association denying that the resolutions were intended to give authority for more than preliminary arrangements. That the committee should, without further and more definite instructions, make any appointments in the organization of the Congress was the grounds of complaint; and it was a secondary objection that they appointed themselves and their friends, including aliens, and excluding representatives from three-fourths of the profession of America.

Now, when the Association found itself in such an anomalous position, its orders and intentions misunderstood, and a privilege never intended to be conveyed, not only usurped, but abused, and the Association thereby placed in an attitude of honoring, above all others, the men who had voluntarily withdrawn from fellowship with them, and had placed themselves in open hostility to the Association, what was expected of it? What should the Association have done?

It was clearly on the defensive, and its dignity and honor were challenged.

Should the American Medical Association quietly submit to what the most indifferent observer must have recognized as an unwarranted assumption of power, and a flagrant abuse of it? Condon such a breach of authority, of trust, of precedent; and, as we said, when urging the Association to assert its rights, "forever let go out of its hands the exercise of one of its highest and most responsible prerogatives—thus establishing a precedent which, justifying all future arrangement committees in ignoring authority, would prove destructive, not only of the honor and dignity of the Association, but of its very existence?" Should the Association, we say, condon the offense, and endorse the action of the committee, or should it do as it did; *protest* in respectful but unmistakable language, and insist upon its supremacy? Had the Association done otherwise than protest against, *not* the appointment of New Code men (primarily), but against the assumption of authority to make any appointments, however acceptable, or otherwise, it certainly would have deserved to be "written down an ass."

The fact that the committee, in the excess of its arrog—*zeal*, we will say, had been so lost to every sense of delicacy, of propriety, and of justice, as to, first, *appropriate to themselves* as many offices as they could absorb, and then give the balance to a few selected friends, instead of considering the large number of medical men in the several States, whose reputation and ability entitled them to recognition, was a *secondary* offense; but, including as it did, the appointment of aliens to the highest, and favorites to *all* the positions, it was *so* offensive as to acquire undue prominence; and the real cause of the difference between the committee and the Association was forgotten; and the gentlemen who felt, and saw that a high-handed outrage had been unblushingly attempted on the entire membership, and who had the courage to denounce it, are now charged with dragging the code question into the discussion, as "a pretext to get themselves into office." When the "new" committee got the opportunity to appropriate the offices, how many did they take? Their modesty and consideration for others, and for decency, presents a strong contrast to the unblushing greed of those who made haste to provide for themselves first, and who, like the kettle, now call the pot "smut."

There is another strange phase of this affair. It is a singular fact that words, by long misuseage, and, often perversion of meaning, come to acquire a signification just the opposite of the meaning, or idea originally intended to be conveyed. This had its origin, no doubt, in what is called "irony." To illustrate: A very homely woman is quite often spoken of now-a-days as "a beauty," (meaning, "she's a fright.") One says in derision, "You are a pretty fellow;" (meaning anything else.) The "leading lights of American medicine," and some of the leading (and misleading) Journals speak in all seriousness of that strange quintescence of egotism, vanity, self-conceit and presumption which prompted the "original committee" to give four appointments to one man—take sixteen out of the twenty-four principal appointments to their own immaculate selves, and to give the other four hundred odd minor places to the northeast corner of American medicine, as "the liberal policy!" "*That broad and liberal policy adopted by the original committee!*" Surely, they do so in a "Pickwickian sense," or in some other sense; or, is it so absurd as to come within the scope of our illustration? To less bigoted minds it appears a most narrow-minded and intensely selfish course, not policy, for there was no "policy" in anything so revolting to a refined sense of justice and fairness.

But, it is asked, "was it *wise* for the American Medical Association to do as it did?" "Was it expedient?" Ah, that is quite a different matter. Perhaps, if it could have been foreseen that these gentlemen were going to act so like that western fellow's chicken that ran in the fire, "without the exercise of any intellectual power," rashly, inconsiderately, and without a reason, (for we submit, they have *never* given an intelligent reason for refusing to participate in the organization of the Congress). If we say, all this fuss and confusion, and illfeeling, and outright misrepresentation; all this accusation and recrimination could have been foreseen, and that it would, ultimately, lead to a crippling of the committee in its work, and, perhaps, to marring the success of the Congress,—the more conservative of the members would, perhaps, have counseled an opposite course; would have said, "better let Billing's committee have it their own way; better swallow the affront and the new-code men, at one gulp, than to make anybody mad." But, no process of reasoning could have foreseen that men, known to be intelligent, and occupying exalted positions, would act more like a disappointed, pouting child, than like rational beings. And, perhaps, it is best that it could not be foreseen ; otherwise the Association *might* have been bullied,brow-beat and *Billings-ed* into submission. For our part, we rejoice that it could not be foreseen ; and we commend the members who had the manliness to speak out in resentment of the imposition !

It is possible, perhaps, to anticipate any reasonable, rational act ; but, who, we say, could have foreseen the dog-in-the-manger spirit that has actuated some twenty-eight out of the four hundred physicians in Philadelphia, and a smaller percentage in three or four other cities, to endeavor, because thwarted in a monstrous attempt to constitute themselves and the new code men of New York *the* medical profession of America, and to ignore the rest of mankind, to resort to *such* means to obstruct the organization of the Congress? Who could have foreseen that the chairman of the immortal committee of eight, after having professed to be satisfied with Speaker Randall's decision that the action of the Association was legal, and after meeting with, and working with, the committee at Chicago, should, within forty-eight hours, go back on them, and join the disgruntled ?

The *right* to do as it did is, we believe, now generally conceded to the Association; hence the question is resolved into one of expediency, "Was it wise? Was it expedient?" But, it will be answered, they did give a reason. They are not willing to admit that it was sympathy with the new code-men; for many of these protestants claim to be "staunch supporters of the code."(?) They claim that the committee, as at present organized, "are not representative men;"—that they are "men of whom we know nothing or too much;"—"men who should not be endorsed," etc.; and lastly, that the new committee are not competent to organize the Congress. The former allegations are made only in spite,and are unworthy of notice. Time will prove the fallacy of the last.

The fact is, most of them are down on Dr. Shoemaker. They say he is unpopular. His election on the committee, by his State delegation,and his election by the committee, as their Secretary, establish the reverse as true. The secret of their opposition to him, in addition to his succees in Philadelphia, despite the small persecution of certain of the mutual admiration clique, springs out of his manly exposition of the Copenhagen racket. That's what's the matter with Shoemaker; but as Father Davis says, "they have not the manliness to acknowledge it."

But enough has been said on this subject. We see by the leading organ of the opposition, the "News," that the decliners have realized the absurdity and the seriousness of the situation, and are now showing some concern as to *how* things can be adjusted! We will surely meet them half way, and in a liberal spirit, entertain any reasonable proposition looking to their participation in the Congress—both they and their new code friends—any proposition that does not compromise the dignity of the Association. While we are not willing to admit, by any means, that the presence or co-operation of those gentlemen, who have "declined," is essential to the holding of a successful Congress, we will admit that it is desirable, and would add eclat to the occasion. An old Darky, being caught between the lines of battle at Manassas, exclaimed: "My God! White folks killin' each other like nothin'—why don't they stop and argify?" We are in favor of ceasing hostilities, and of letting reason resume sway; and no doubt some basis of settlement may be suggested—alike acceptable and honorable to all. There can be no doubt that the reputable profession of America are entitled to participation in the Congress, whether they be members of the Association or not, but it is absurd to expect the American Medical Association to appoint to the highest offices those who have openly repudiated its Code of Ethics. The good old minister said he was willing to receive repentent sinners back into the fellowship of the church, but he be blessed if he favored making deacons of them. So we would consent to new-code men participating in the Congress, but not as delegates from, nor *quassi* representatives of the American Medical Association, so long as they are unrepentent sinners.

Be the fault whose it may whereby the present unfortunate state of affairs has been brought about, (neither party will acknowledge it,) let the quarrel stop, and let an endeavor be made to harmonize all conflicting interests. It is preposterous, however, to ask or expect the Association to rescind its action; it cannot receed with honor from the position taken. Right is one thing, expediency is quite another. Who will then be the peace-maker? Who the good Samaritan? Who the Moses to guide these medical children out of the wilderness of wind and words, and the mire of misunderstanding and misrepresentation, and guide them to the promised land of the International Congress, and make things "as serene as a mother-in-law's life?"

We have already, in our last issue indicated the right course: What are called new-code men are those who left the organization of rational medicine, on a misinterpretation of the code. Now that it has been demonstrated to the world *to have been* a misinterpretation, and those gentlemen officially assured of it by the Association, the doors are once more open to them to return to the fold—and they should do so without hesitation. Thus all differences would be settled; and then the American Medical Association could, consistently and with honor, and without stultifying itself, appoint them to high offices in the Congress. We would then, perhaps, be willing to "make deacons of some of them."

